# Supplementary material for: Proportion of foetal and placental implantation abnormalities in Madagascar: A cross-sectional study of 35,919 women at public-sector primary healthcare facilities in central and southern Madagascar, 2017–2020
Source: PLoS One. 2024 Dec 31;19(12):e0311918. doi: 10.1371/journal.pone.0311918 (PMC11687778; doi:10.1371/journal.pone.0311918)
Supplement: S1 File — (DOCX) [file pone.0311918.s001.docx]

**Supplementary File 1**

**Proportion of foetal and placental abnormalities in Madagascar: a cross-sectional study of 35,919 women at public-sector primary healthcare facilities in central and southern Madagascar, 2017-2020**

| **Number of revisits** | **Placenta previa I** | **Placenta previa II** | **Placenta previa III** | **Placenta previa, no categorisation** | **Missing information** | **No placenta previa** |
| --- | --- | --- | --- | --- | --- | --- |
| 0 | 556 | 57 | 6 | 114 | 783 | 31894 |
| 1 | 94 | 8 | 0 | 19 | 36 | 4725 |
| 2 | 15 | 2 | 0 | 3 | 7 | 420 |
| 3 | 1 | 0 | 0 | 0 | 1 | 14 |

| **Number of revisits** | **Number of foetus(es) = 1** | **Number of foetus(es) = 2** | **Number of foetus(es) = 3** | **Missing information** |
| --- | --- | --- | --- | --- |
| 0 | 32246 | 349 | 4 | 753 |
| 1 | 4792 | 59 | 0 | 22 |
| 2 | 437 | 10 | 0 | 0 |
| 3 | 13 | 3 | 0 | 0 |

| **Number of revisits** | **Oligoamnios** | **Polyhydramnios** | **Normal amniotic fluid** |
| --- | --- | --- | --- |
| 0 | 25 | 173 | 32259 |
| 1 | 4 | 28 | 4748 |
| 2 | 1 | 3 | 433 |
| 3 | 0 | 0 | 16 |

**Supplementary Table 1.** Distribution of foetal and placental abnormalities and the number of ultrasound examinations in our study sample. The analyses revealed no significant correlation between the number of foetuses, amniotic fluid disorders, or placental and foetal pathologies and the number of ultrasound visits. Pearson’s chi-square test revealed a significant correlation between the grade of placenta praevia and the number of revisits (p <0.01).
