# Supplementary material for: Proportion of foetal and placental implantation abnormalities in Madagascar: A cross-sectional study of 35,919 women at public-sector primary healthcare facilities in central and southern Madagascar, 2017–2020
Source: PLoS One. 2024 Dec 31;19(12):e0311918. doi: 10.1371/journal.pone.0311918 (PMC11687778; doi:10.1371/journal.pone.0311918)
Supplement: Attachment — Submitted filename: Reply to reviewers 25052024.docx [file pone.0311918.s002.docx]

Berlin, May 25^th^ 2024

Author's response to peer review

Paper ID: PONE-D-24-05563

Title: Proportion of foetal and placental abnormalities in Madagascar: a cross-sectional study of 35,919 women at public-sector primary healthcare facilities in central and southern Madagascar, 2017-2020

Version: Response to peer review version 1.0

Dear Editor,

First, we express our gratitude to the reviewers for their valuable and constructive input. We thank the reviewers for the positive feedback on our article and for recognising the value in presenting data from an understudied region. We are happy to hear that our paper is potentially acceptable for publication in your journal.

Enclosed is a detailed response addressing all reviewer and editor comments. We have incorporated the suggested changes, clearly indicated through track changes in the accompanying manuscript.

We look forward to your feedback and thank you for your consideration.

Dr Julius Emmrich

| Reviewer #1 |  |
| --- | --- |
| The introduction reads well but given that your title and focus of the paper if on foetal and placental anomalies, it would be good to have a paragraph on foetal anomalies as well some literature on placenta, particularly because the placenta is critical for foetal development. | We thank the reviewer for highlighting this point and agree that the introduction of the paper would benefit from more detail on the importance of foetal and placental anomalies in the introduction. We have therefore added the following section to lines 71 to 79:  “Identifying foetal and placental abnormalities during pregnancy is essential to identify potential complications during gestation or delivery. Foetal abnormalities, e.g., congenital malformations can necessitate additional provisions for delivery care, for example by requiring a Caesarean Section. Equally, placental abnormalities such as placenta praevia or placenta accreta may require additional precautions during delivery. Placental abnormalities may affect the development of the foetus in utero, requiring additional antenatal or postnatal care and supervision. As such, accurate information on placental and foetal abnormalities that can be obtained from obstetric ultrasounds is vital for adequate antenatal support, delivery planning and reducing maternal and neonatal mortality.” |
| It is also important to highlight, briefly the importance of obstetric ultrasound screenings and how cases with adverse events are reduced. There is some information on this in line 77 but a suggestion to expound briefly on this. This will broaden the readership of this interesting article. | We thank the reviewer for highlighting this point and have integrated a more detailed description of the value of antenatal ultrasounds in reducing adverse events in lines 71 to 79 as highlighted above. |
| Methods Study setting Kindly provide a reference for the statement from line 114 – 117 providing the statistics on the number of women who attend antenatal care visits within these remote regions. | We apologise for the oversight of failing to include a citation in the sentence. We have now rectified our mistake. |
| Study design Line 129 – 131 (Line beginning with: Following ultrasound exams…) can be moved to the Data Analysis section. This is because this refers to the reports where the data was obtained from. | We agree with the reviewer that this sentence may be better placed in the section relating to data analysis and we have moved it to lines 210ff. |
| Study sample Line 222 - 229 – please consider summarizing this in a flow diagram. It will be much easier for the reader to understand the numbers. | We thank the reviewer for raising this issue and noticed the lack of clarity in this paragraph. In particular, these numbers do not represent exclusion criteria, so we rewrote the paragraph. More importantly, following the reviewer’s recommendation to outline the timing of follow-up visits, we identified 20 women with twin pregnancies therefore had multiple data entries in one visit, instead of having multiple visits. We have therefore corrected relevant numbers in **(**a) this paragraph, (b) the section “Timing of ultrasound exams during pregnancy”, and (c) Table 1 accordingly.   a) Lines 252 to 259 now read:   “A total of 38,688 ultrasound exam reports (data entries) were collected, from which we identified 33,332 individuals who had one single ultrasound examination and 2,587 individuals who had more than one ultrasound examination. Accordingly, an individual may have had multiple data entries depending on the number of ultrasound examinations she had. Therefore, the 38,688 data entries belonged to 35,919 women. Of these 38,688 reports, 492 lacked data on the time of the first ultrasound, and 827 lacked data on placenta location. Of these 35,919 women, 762 had missing information on the number of foetuses. Of all foetuses examined, information on amniotic fluid volume was missing in 1,012 cases.”   b) We have added the following details to the section “Timing of ultrasounds exams during pregnancy (lines 352 to 371):   For the 2,587 women who had more than one ultrasound examination, we also analysed when the follow-up visits occurred. Among these 2,587 women, inconsistencies between date of facility visit and trimester (e.g., earlier date is associated with a later trimester) or missing information were observed in data from 19 women, leading to exclusion from this analysis. Of the 2,568 women included, 2,418 had two ultrasound examinations (1 revisit; 94.16%), 147 had three ultrasound examinations (2 revisits; 5.72%), and 3 had four ultrasound examinations (3 revisits; 0.12%).  Among the 2,418 women who had two ultrasound examinations, a total of 256, 1433, and 729 first examinations occurred in the first, second, and third trimesters, respectively, and the follow-up occurred in the first trimester in 6 women, in the second trimester in 150 women, and in the third trimester in 2262 women. Of the 147 women who had three ultrasound examinations, 35 had their first examination in the first trimester, 95 had their first examination in the second trimester, and 17 had their first examination in the third trimester. The first follow-up was in the second trimester in 48 women and in the third trimester in 99 women, respectively. A total of 2 women had the second follow-up in the second trimester, and 145 women in the third trimester. Of the 3 women who had four ultrasound examinations, 2 had their first examination in the first trimester and 1 in the second trimester. The first follow-up occurred was in the second trimester for one woman, and in the third trimester for 2 women. The second and third follow-ups were all in the third trimester. |
| Analysis Line 241 - What was the criteria used to define placenta pathology? | We agree with the reviewer that the description of the categories in this paragraph lacked clarity. We have amended the wording as follows:  “Secondly, we estimated the proportion of four groups of foetal and placental abnormalities, **defined as** (i) abnormal placenta position, (ii) number of foetuses above one, (iii) amniotic fluid disorders, and (iv) other placental and foetal pathologies **respectively (e.g., placental adherence disorders,** foetal malformations and death).” |
| Kindly clarify line 243 (Line starting with: For all parameters..) as this is not clear who exactly was included in the descriptive statistics. The section is confusing as it is not clear who the 35919 and 38688 represent and why different participants were used in the analysis and there was no common denominator. Kindly provide some clarity. | We apologize for the lack of clarity, and have included detailed descriptions of how these numbers were determined. We hope this together with the description of study samples could make everything clear. The paragraph now reads:  “A total of 38,688 ultrasound exam reports (data entries) were collected, from which we identified 33,332 individuals who had one single ultrasound examination and 2,587 individuals who had more than one ultrasound examination. Accordingly, an individual may have had multiple data entries depending on the number of ultrasound examinations she had. Therefore, the 38,688 data entries belonged to 35,919 women. Of these 38,688 reports, 492 lacked data on the time of the first ultrasound, and 827 lacked data on placenta location. Of these 35,919 women, 762 had missing information on the number of foetuses. Of all foetuses examined, information on amniotic fluid volume was missing in 1,012 cases.” |
| Results Was there a correlation between region (rural vs urban) and number of ultrasounds | There was no significant correlation between the region and the number of ultrasounds in our study sample. However, as we highlighted in lines 349 to 351, there was a significant difference in the timing of the ultrasound visits between the two populations:  “The proportion that received a scan in the third trimester was significantly higher among women from rural backgrounds (71.33%, 23,352/32,740) than from urban backgrounds (53.77%, 3,198/5,948; p-value: <0.01).” |
| For the women who had >1 ultrasound, which trimesters were they categorized under for their first and follow up visits? | We thank the reviewer for this recommendation and believe the information is highly relevant to the aims of the manuscript. Two new paragraphs highlighting these results have been added to the section “**Timing of ultrasound exams during pregnancy**”:  “For the 2,587 women who had more than one ultrasound examination, we also analysed when the follow-up visits occurred. Among these 2,587 women, inconsistencies between date of facility visit and trimester (e.g., earlier date is associated with a later trimester) or missing information were observed in data from 19 women, leading to exclusion from this analysis. Of the 2,568 women included, 2,418 had two ultrasound examinations (1 revisit; 94.16%), 147 had three ultrasound examinations (2 revisits; 5.72%), and 3 had four ultrasound examinations (3 revisits; 0.12%).  Among the 2,418 women who had two ultrasound examinations, a total of 256, 1433, and 729 first examinations occurred in the first, second, and third trimesters, respectively, and the follow-up occurred in the first trimester in 6 women, in the second trimester in 150 women, and in the third trimester in 2262 women. Of the 147 women who had three ultrasound examinations, 35 had their first examination in the first trimester, 95 had their first examination in the second trimester, and 17 had their first examination in the third trimester. The first follow-up was in the second trimester in 48 women and in the third trimester in 99 women, respectively. A total of 2 women had the second follow-up in the second trimester, and 145 women in the third trimester. Of the 3 women who had four ultrasound examinations, 2 had their first examination in the first trimester and 1 in the second trimester. The first follow-up occurred was in the second trimester for one woman, and in the third trimester for 2 women. The second and third follow-ups were all in the third trimester.” |
| Was the number of ultrasound exams associated with any of the factors in Table 2? | We agree with the reviewer that the relationship between the number of ultrasound exams and the foetal and placental abnormalities merits further analysis. We have run the additional analysis and have made the following changes to a) lines 303 to 305 of the methods section, b) lines 399 to 402 of the results section, and c) lines 478 to 485 of the discussion section. The respective sections now read as follows.  a) lines 303 to 305:  “We equally used Pearson's chi-squared test (for categorical data) and Spearman rank-order correlation coefficient (for continuous data) to analyse correlations between foetal and placental abnormalities and the number of ultrasound examinations”  b) lines 399 to 402:  “Additional analyses showed no significant correlation between the number of foetuses, amniotic fluid disorders, or placental and foetal pathologies and the number of ultrasound visits. Pearson’s chi-square test revealed a significant correlation between the grade of placenta praevia and the number of revisits (p <0.01).”   c) lines 478 to 485:   “Additionally, our study shows that there was no significant correlation between the number of ultrasounds visits and foetal and placental abnormalities that had been identified during obstetric ultrasounds, apart from the grade of placenta praevia. This is worrying as several of the pathologies identified should be followed up through repetitive ultrasound examinations in pregnancy. Our findings indicate that women in Madagascar with foetal and placental abnormalities do not access repetitive ultrasound examinations in such cases. Further indicate should be conducted to identify the reasons behind these healthcare seeking behaviours.” |
| Table 1 if Missing is 0, it can be excluded from the table | We thank the reviewer for highlighting this point and have amended Table 1 accordingly. |

| Reviewer #2 |  |
| --- | --- |
| The paper is overall well organized and well written with appropriate style and language. The flow is logical and well structured mostly and the chosen style is consistent throughout. The tables presented are straightforward although graphs could have been used to make the paper more impressive. What makes the paper interesting is that it covers a part of Sub-Saharan Africa, Madagascar which has a high burden of increased maternal and neonatal mortality. The study analyzed 38,685 ultrasound screening reports which is a very significant number along with the demographic characteristics. | We thank the reviewer for this positive feedback on our paper. |
| However, the aims and objectives of the study are not well defined. They mention that strategies aimed at reducing maternal and neonatal mortality are challenging to define and implement. But a clear aim of the study is not clear. (Major Issue). | We agree with the reviewer that the aim of our study could have been more clearly stated. We have therefore revised lines 115 to 121 of the manuscript as follows:  “The aim of this study is to describe the demographic characteristics of a large group of pregnant women who utilized obstetric ultrasound services provided through an NGO. Specifically, we aim to determine the prevalence of foetal and placental abnormalities in this study sample and to assess the timing and frequency of ultrasound examinations among these women. We expect these data to guide policymakers and program implementers in refining the design, implementation, and resource allocation of maternal health initiatives in this setting.” |
| The limitations and strengths of the study have been discussed very clearly and that is worth mentioning. | We thank the reviewer for this positive feedback on our discussion section. |
| With regards to the results obviously, most of the results cannot be accurately interpreted as the ultrasounds were not performed at the desired gestational ages and the fetal congenital abnormalities have been mentioned very briefly and with no clear description which is interpreted from the data. The authors however should be credited for their job with the best of what could be expected from the data obtained from underdeveloped rural areas of Sub Saharan Africa. | Thank you for your observations regarding the limitations of our results. We acknowledge the challenges in interpreting the data due to the varied gestational ages at which the ultrasounds were performed as well as incomplete and missing data. We aimed to clarify these descriptions in the revised manuscript within the constraints of the data available. |
