# Supplementary material for: Proportion of foetal and placental implantation abnormalities in Madagascar: A cross-sectional study of 35,919 women at public-sector primary healthcare facilities in central and southern Madagascar, 2017–2020
Source: PLoS One. 2024 Dec 31;19(12):e0311918. doi: 10.1371/journal.pone.0311918 (PMC11687778; doi:10.1371/journal.pone.0311918)
Supplement: Attachment — Submitted filename: Reply to reviewers 12072024.docx [file pone.0311918.s003.docx]

Berlin, July 19^th^ 2024

Author's response to peer review

Paper ID: PONE-D-24-05563

Title: Proportion of foetal and placental abnormalities in Madagascar: a cross-sectional study of 35,919 women at public-sector primary healthcare facilities in central and southern Madagascar, 2017-2020

Version: Response to peer review version 2.0

Dear Editor,

We would like to thank the reviewers for their insightful and constructive feedback on our previous resubmission. In the enclosed document, we have provided detailed responses to all comments from the reviewers in this second round of revisions. The suggested revisions have been incorporated into the manuscript, with changes marked using track changes.

We look forward to your feedback and thank you for your consideration.

Dr Julius Emmrich

| Reviewer #1 |  |
| --- | --- |
| Line 71 - 79: The paragraph included on foetal and placental abnormalities requires supporting references. Kindly include recent references to support the statements provided. | We agree with the reviewer that the previous version of the paragraph lacked references. We have included relevant references into the paragraph as follows:  *Obstetric ultrasound is a non-invasive, sensitive, accurate and cost-effective imaging technique for the detection and diagnosis of congenital abnormalities, obstetric pathologies, and complications of pregnancy. The use of obstetric ultrasound is proliferating in resource-constrained settings due to its increased affordability, availability, portability, and durability* ***[1].*** *Identifying foetal and placental abnormalities during pregnancy is essential to identify potential complications during gestation or delivery* ***[2].*** *Foetal abnormalities, e.g., congenital malformations can necessitate additional provisions for delivery care, for example by requiring a Caesarean Section* ***[3].*** *Equally, placental abnormalities such as placenta praevia or placenta accreta may require additional precautions during delivery* ***[2].*** *Placental abnormalities may affect the development of the foetus in utero, requiring additional antenatal or postnatal care and supervision* ***[4].*** *As such, accurate information on placental and foetal abnormalities that can be obtained from obstetric ultrasounds is vital for adequate antenatal support, delivery planning and reducing maternal and neonatal mortality.  References: 1. Sippel S, Muruganandan K, Levine A, Shah S: Use of ultrasound in the developing world. Int J Emerg Med 2011, 4(72) 2. Goley SM, Sakula-Barry S, Adofo-Ansong N, et al. Investigating the use of ultrasonography for the antenatal diagnosis of structural congenital anomalies in low-income and middle-income countries: a systematic review. BMJ Paediatr Open. 2020;4(1):e000684. Published 2020 Aug 20. doi:10.1136/bmjpo-2020-000684*  *3. Wataganara, T., Grunebaum, A., Chervenak, F. & Wielgos, M: Delivery modes in case of fetal malformations. Journal of Perinatal Medicine 2017, 45(3): 273-279. https://doi.org/10.1515/jpm-2015-0364*  *4. Kim, E.T., Singh, K., Moran, A. et al. Obstetric ultrasound use in low and middle income countries: a narrative review. Reprod Health 15, 129 (2018). https://doi.org/10.1186/s12978-018-0571-y* |
| Line 115 - 121: Suggestion to use past tense, the aim of this study was to.. as you are now reporting on the findings | We thank the reviewer for this suggestion and have adapted the tense of the paragraph accordingly.  *The aim of this study* ***was*** *to describe the demographic characteristics of a large group of pregnant women who utilized obstetric ultrasound services provided through an NGO. Specifically, we* ***aimed*** *to determine the prevalence of foetal and placental abnormalities in this study sample and to assess the timing and frequency of ultrasound examinations among these women. We expect these data to guide policymakers and program implementers in refining the design, implementation, and resource allocation of maternal health initiatives in this setting.* |
| Line 141: Include a reference for Open Street Map as per the Journal guidelines | We have included a reference of Open Street Map in the bibliography of the paper. |
| Line 248: Study sample can read study cohort | We thank the reviewer for this suggestion and have changed the subtitle from “study sample” to “study cohort” |
| For the study sample section it will be easier to understand the numbers if depicted in a flow diagram. Kindly consider including one so that the readers can better understand the flow and final selection of study participants | We thank the reviewer for the suggestion of including a flow diagram. We have uploaded such a diagram with our submission and added the following description of the diagram to the manuscript.    *Figure 2: Study cohort*  *Figure 2. Depiction of study sample of women included in the study between July 1, 2017, and September 30, 2020, in urban (Analamanga) and rural (Atsimo-Andrefana, Anosy, and Androy) Madagascar.* |
| Line 283: there is a bracket close but not sure where the opening bracket is | The opening bracket for the closing bracket after the end of the sentence can be found after “respectively”, highlighted in bold below:  *Secondly, we estimated the proportion of four groups of foetal and placental abnormalities, defined as (i) abnormal placenta position, (ii) number of foetuses above one, (iii) amniotic fluid disorders, and (iv) other placental and foetal pathologies,* ***respectively (****e.g., placental adherence disorders, foetal malformations, and death).* |
| Line 302 and 303: The correlations can be depicted as graphs as this is briefly mentioned in Line 398 - 401. It is a important finding; and later discussed in line 477-480. | We thank the reviewer for recognising the importance of these findings. As most of the analyses conducted involved categorical variables, we perceived tables as a more adequate method of depicting these analyses. We have uploaded these tables as *Supplementary Table 1* and included them here below. |

| **Number of revisits** | **Placenta previa I** | **Placenta previa II** | **Placenta previa III** | **Placenta previa, no categorisation** | **Missing information** | **No placenta previa** |
| --- | --- | --- | --- | --- | --- | --- |
| 0 | 556 | 57 | 6 | 114 | 783 | 31894 |
| 1 | 94 | 8 | 0 | 19 | 36 | 4725 |
| 2 | 15 | 2 | 0 | 3 | 7 | 420 |
| 3 | 1 | 0 | 0 | 0 | 1 | 14 |

| **Number of revisits** | **Number of foetus(es) = 1** | **Number of foetus(es) = 2** | **Number of foetus(es) = 3** | **Missing information** |
| --- | --- | --- | --- | --- |
| 0 | 32246 | 349 | 4 | 753 |
| 1 | 4792 | 59 | 0 | 22 |
| 2 | 437 | 10 | 0 | 0 |
| 3 | 13 | 3 | 0 | 0 |

| **Number of revisits** | **Oligoamnios** | **Polyhydramnios** | **Normal** |
| --- | --- | --- | --- |
| 0 | 25 | 173 | 32259 |
| 1 | 4 | 28 | 4748 |
| 2 | 1 | 3 | 433 |
| 3 | 0 | 0 | 16 |

Supplementary Table 1. Distribution of foetal and placental abnormalities and the number of ultrasound examinations in our study sample. The analyses revealed no significant correlation between the number of foetuses, amniotic fluid disorders, or placental and foetal pathologies and the number of ultrasound visits. Pearson’s chi-square test revealed a significant correlation between the grade of placenta praevia and the number of revisits (p <0.01).

| Reviewer #2 |  |
| --- | --- |
| The manuscript has been revised and queries have been answered, but the limitation of the manuscript is quite big. The results drawn are not representing the exact fetal and placental pathology of the population due to the various reasons described in the limitations the most highlighted being the timing of ultrasound which is mainly 3rd trimester.  Similarity of results with other Sub Sahara Regions may not be an accurate criteria of comparison as similar limitations may have been encountered in those regions.  Hence, the authors should strongly recommend changing the policy of access to good quality ultrasound facility for all women in the first and second trimesters mainly and to emphasize the NGO's and governments to strongly implement upon the introduction of ultrasound in the first and second trimesters.  This will enable to get a more accurate data for the incidence of fetal and placenta pathologies in the concerned population. | Thank you for your thoughtful feedback and for acknowledging the revisions made to our manuscript. We appreciate your concerns regarding the limitations of our study, particularly the timing of the ultrasound assessments predominantly being in the third trimester.  We agree that the timing of ultrasound examinations can significantly influence the accuracy of detecting fetal and placental pathologies. Our study acknowledged this limitation and we understand that it may affect the generalizability of our findings. We have added an additional sentence regarding the comparability of our findings to lines 491ff. of the discussion section:  **“We do acknowledge however, that this may not be an accurate comparison, as studies conducted in similarly resource-constrained settings in SSA may have been affect by similar limitations.”**  However, as we describe in our manuscript, we believe our findings, describing obstetric pathologies in a severely understudied region, are of relevance for policy-makers and implementers alike.  As suggested by the reviewer, we have added a recommendation on accessing ultrasounds earlier in pregnancy to the conclusions section of our manuscript, which now reads:  *“Our data show that obstetric ultrasound in a resource-restricted setting can lead to the detection and diagnosis of high-risk obstetric conditions including placental abnormalities, malpresentation of the foetus, and multiple gestations, which are highly associated with maternal and foetal morbidity and mortality. In stark contrast to current WHO recommendations, our findings suggest that universally accessible obstetric ultrasound is commonly and mostly utilised by pregnant women, especially those from rural, resource-constrained settings, during the third trimester of pregnancy.* ***This finding highlights the need to improve policy and practice in Madagascar to improve access to obstetric ultrasounds, particularly during the first and second trimesters.”*** |
