# Supplementary material for: Proportion of foetal and placental implantation abnormalities in Madagascar: A cross-sectional study of 35,919 women at public-sector primary healthcare facilities in central and southern Madagascar, 2017–2020
Source: PLoS One. 2024 Dec 31;19(12):e0311918. doi: 10.1371/journal.pone.0311918 (PMC11687778; doi:10.1371/journal.pone.0311918)
Supplement: Attachment — Submitted filename: Response to Reviewers_PLOS_16092024.docx [file pone.0311918.s004.docx]

Berlin, September 24^th^, 2024

Author's response to peer review

Paper ID: PONE-D-24-05563

Title: Proportion of foetal and placental abnormalities in Madagascar: a cross-sectional study of 35,919 women at public-sector primary healthcare facilities in central and southern Madagascar, 2017-2020

Version: Response to peer review version 3.0

Dear Editor,

We appreciate the reviewers' feedback on our previous resubmission. In the attached document, we have provided detailed responses to all of the reviewers' comments in this third round of revisions. We have incorporated the suggested changes into the manuscript, with all revisions clearly marked using track changes.

We look forward to your feedback and thank you for considering our manuscript.

Dr Julius Emmrich

| Reviewer #3 |  |
| --- | --- |
| Due to the manuscript's localized focus, lack of originality, and insufficient contribution to the broader field, it is recommended for rejection. The study does not present new knowledge or significantly advance the existing body of literature, which is a critical criterion for publication in this journal | We respectfully disagree with the reviewer's assessment. for the following reasons:   1. Large dataset: Our study analyses 35,919 pregnancies, making it the largest and most comprehensive obstetric ultrasound dataset from Madagascar and one of the few at this scale in Sub-Saharan Africa. It provides robust maternal health data in a severely resource-constrained setting. 2. Global relevance: While focused on Madagascar, our findings on foetal malpresentation, placental abnormalities, and late obstetric screening have broad implications for similar settings in other low- and middle-income countries. In particular, our study exposes stark discrepancies between current WHO recommendations and real-world practices in LMICs. 3. Policy implications: By documenting the high prevalence of late-pregnancy ultrasounds in contrast to WHO recommendations for early screening, our study provides evidence-based observations for health policy reform. These findings are critical for improving maternal and neonatal care strategies in Madagascar and similar resource-limited settings globally. 4. Peer recognition: Five previous reviewers have recommended this manuscript for publication in PLOS One, acknowledging its scientific merit and contribution to the field. |

| Reviewer #4 |  |
| --- | --- |
| The world health recommendation of ultrasound scan before 24 weeks of gestation is to screen for congenital abnormalities, in the current manuscript late ultrasound scans were performed in preparation for delivery - the two uses of the ultrasound scan in pregnancy (screening for congenital anomalies vs preparation for delivery don't match) | We agree with the reviewer that there is a discrepancy between the recommended timing of ultrasounds by the WHO and the timing when most women in our study sample accessed ultrasound services.  As this is an observational study however that draws on existing data from an NGO intervention, there was no possibility to influence the timing of ultrasounds in the study sample. Despite mostly accessing ultrasound scans late in pregnancy, for most women in our sample these were still their first or only obstetric ultrasound examinations. Hence screening for congenital abnormalities where still conducted and reported even if the examination was conducted after 24 weeks gestation. |
| Line 49-50: The proportion of malposition is reported and breakdown of those malapositions are presented as proportions of the total number of malpositions found. I would rather the authors present the constituent malpositions as proportions of the total number of malpositions found. Otherwise teh denominators get confusing for the reader. | We agree with the reviewer that the previous reporting of our findings was not clear and have amended the denominator accordingly. Lines 48ff now read:  *“Foetal malpresentation at 36 weeks of gestation or later was diagnosed in 5.48% (176/3,211) of women with the breech presentation being most common (breech* ***72.73%, 128/176;*** *transverse* ***15.34%, 27/176****; mobile* ***9.01%, 16/176****; oblique* ***2.84%, 5/176).****”* |
| In the methods section the authors should describe what happened to participants with more than one ultrasound scan - say early and late ultrasound scan - which of the two was analysed? How was teh selection for analysis arrived at? | We would like to draw the reviewer’s attention to lines 225 to 239 of the manuscript where we explain how we identified and matched the reports of study participants who received more than one ultrasound examination, as well as lines 268 to 283 of the manuscript where we detail our approach in analysing the data from women with multiple ultrasound visits.  Briefly, all data from both visits were analysed in the paper, and the denominator was either the number of total patients enrolled in the study (when analysing ultrasound findings that do not change over time, e.g. the number of foetuses) of the total number of ultrasound examinations done, including re-visits (when analysing ultrasound findings that can change over time, e.g. foetal presentation. |
| In the results sections, authors report 492 participants has missing data - which type of data was this? Was it demographic characteristics, distance from the health facility, or reports missing critical information? It would be more informative for the reader if this missing data is defined. | We would like to draw the reviewer’s attention to lines 247ff. of the results section, where we detail the missing data of our study participants:  *“Of these 38,688 reports, 492 lacked data on the time of the first ultrasound, and 827 lacked data on placenta location. Of these 35,919 women, 762 had missing information on the number of foetuses. Of all foetuses examined, information on amniotic fluid volume was missing in 1,012 cases.”* |
| In the discussion, line 328 - over 35,000 women; I would rather the authors re-state their total sample size. | We agree with the reviewer and have changed the “over 35,000 women” to “35,919 women” in line 391 of the manuscript. |

| Reviewer #5 |  |
| --- | --- |
| Reviewer #5: Thank you for your understanding despite my delay in trying to go through the corrected work and initial reviewers suggestions, I found that the authors have made substantial improvement in the manuscript compared to the initial submission. Most of the corrections were done. | We thank the reviewer for their time and effort in providing us with valuable feedback on our work and are happy that they recognise the effort we have made in improving our manuscript according to the feedback received. |

| Reviewer #6 |  |
| --- | --- |
| 1. Line 1 "Tittle) from the scientific point of view, Placental abnormalities refer to any deviations from the normal structure or function of the placenta during pregnancy. These can be classified into several types:  Structural Anomalies: These include conditions like a succenturiate lobe (an extra lobe of the placenta) or velamentous cord insertion (where the umbilical cord attaches to the fetal membranes rather than the placenta itself). Implantation Anomalies: These include placenta accreta (where the placenta attaches too deeply into the uterine wall) and placenta previa (where the placenta covers the cervix). Functional Anomalies: These include placental insufficiency, where the placenta does not function properly, leading to inadequate supply of oxygen and nutrients to the fetus.  among these, you only focused on implantation Anomaly. so it is better if you modify your title and objectives accordingly. | We thank the reviewer for highlighting this important point and agree that we only focused our analysis on one dimension of placental abnormalities. We have therefore amended the title, short title, abstract and manuscript, to reflect “placental implantation abnormalities” instead of “placental abnormalities” more broadly. |
| I noticed that the research question might not have been clearly addressed. Could you provide a single answer to the question: What is the proportion of fetal and placental abnormalities? Please ensure the conclusion of the abstract also reflects this information. | We agree with the reviewer that the abstract in it’s current form lacked a concise answer to the research question. We have therefore added the following sentence to lines 52 to 53 of the abstract:  *“In a total of 1,284 cases (3.65%) a foetal of placental implantation abnormality was found.”*  We have further amended the conclusions section of the abstract to reflect this statement:  *“The proportion of foetal and placental* ***implantation*** *abnormalities detected by obstetric ultrasound* ***was 3.65% of all cases in our study,*** *aligning with findings from other countries in sub-Saharan Africa.”* |
| Line 62: Which keywords best describe your objectives? I couldn’t find keywords that reflect your title. | We agree with the reviewer that our current selection of key words do not reflect our study’s title.  We have therefore added the following keywords to the list:  *“Madagascar; foetal abnormalities; placental implantation abnormalities”* |
| Lines 65-69: The paragraph does not support your research question. Your focus is on identifying fetal-placental abnormalities, not the importance of obstetric ultrasound. For instance, if there were other mechanisms or technologies to identify fetal-placental abnormalities, discussing ultrasound would be unnecessary. sPlease rewrite the background section accordingly. | Thank you for your thoughtful comment. While we understand the point raised, we feel that the paragraph, though not directly addressing the research question, is important in providing context. It helps to underscore the relevance of our study, particularly by illustrating the growing use and significance of obstetric ultrasound in low-resource settings, also ensuring the comparability of our findings to other similar environments. For this reason, we would prefer to keep the paragraph as it is. |
| Method: Some mothers visited and had ultrasound scans more than twice. Does this make the study cross-sectional or cohort? What is your perspective? | In a cross-sectional study, data are collected at a single point in time, which aligns with the design of our study analyzing ultrasound screenings over a specific period (2017-2020).  Although some women had multiple ultrasound scans, this does not change the design of our study. The purpose of follow-up ultrasounds is typically to monitor or verify conditions detected in initial scans, but the overall structure of our study remains a snapshot of conditions during the study period. The fact that some participants had more than one ultrasound does not make the study a cohort study, which would require following individuals prospectively over time to observe outcomes.  Thus, despite the repeated ultrasounds for some mothers, the study remains cross-sectional because its main objective is to provide a prevalence estimate during a defined period. We did not focus on long-term outcomes or changes over time. |
| What is the outcome variable in this context? How did you use the chi-square test? What are the independent variables, and how many were tested? These points were unclear to me. | We thank the reviewer for highlighting that the previous description of our use of the Chi-square test lacked clarity.  We have added the following detail to lines 288ff of the manuscript:  *“Specifically, the chi-square test was applied to categorical variables (for example, number of examinations received vs. presence of placenta previa) summarised as a contingency table to understand whether there was a relationship between the two categorical variables."* |
| Lines 299-300 seem more like a discussion or personal opinion. | We agree with the reviewer that the sentence in question appears to be more adequate for a discussion section. We have therefore removed it from the results section. |
| In this paper, I didn't capture clear outcome variable, which is why the authors discussed all the findings. Even it is too long to read and understand. Please rewrite the discussion again. | We agree with the reviewer that from the previous presentation, it was not clear what the main outcome of our study was, and which were subordinate findings. We have therefore restructured the discussion section to report and discuss on the findings relevant to our main research question, the proportion of foetal and placental implantation abnormalities, first. Accordingly, lines 391 to 403 now read as follows:  *“Using data collected from over* ***35,919*** *women, we estimated the proportion of foetal and placental* ***implantation*** *abnormalities among pregnant women attending obstetric ultrasounds at public-sector healthcare facilities in Madagascar.* ***Less than 10% of women in our sample were diagnosed with a potential complication during pregnancy. Foetal malposition was present in 5.48% (176/3,211) of cases and other abnormalities of placental implementation or the foetus occurred in a total of 3.65% (1,284/35,919) of all ultrasound examinations.***  ***Our study further revealed two subordinate but relevant findings.*** *First, most women in our sample came from rural areas. Secondly, less than 10% of women in our sample were diagnosed with a potential complication during pregnancy. Foetal malposition, breech position and abnormal position of the placenta were the most common findings. Lastly, most obstetric ultrasounds were conducted in the third trimester of pregnancy, especially among women from rural backgrounds.”* |
| the conclusion of these findings is unclear. It is necessary to determine whether the proportion is high, acceptable, or low to understand the implications. Lines 504-510 do not reflect the primary objectives of the authors. | We agree with the reviewer that the conclusion sections as it was did not reflect essential information on our primary research objective. We have revised the Conclusion section accordingly. It now reads as follows:  *“Our data show that obstetric ultrasound in a resource-restricted setting can be used for the detection of high-risk obstetric conditions including placental implantation abnormalities, malpresentation of the foetus, and multiple gestations, which. In our sample, these conditions where present in less than 10% of cases and thus as common as in other countries in Sub-Saharan Africa. As a secondary finding, our study showed that accessible obstetric ultrasound is commonly and mostly utilised by pregnant women during the third trimester of pregnancy, especially those from rural, resource-constrained settings, during the third trimester of pregnancy. This is in n stark contrast to current WHO recommendations and highlights the need to ammend policy and practice in Madagascar to improve access to obstetric ultrasounds, particularly during the first and second trimesters.”* |
| What does the author, Prof. Dr. Dr., mean? Is there a recognition of dual doctorates in Madagascar? | Prof. Dr. Dr. Bärnighausen holds two doctorate degrees, one in history of medicine from the University of Heidelberg, and one in Population and International Health from the Harvard T.H. Chan School of Public Health at Harvard University. |
| Authors have omitted the declaration section, which includes crucial components such as the source of funding and other pertinent information. | As per PLOS One submission requirements, we have not included the declarations section within the manuscript but have uploaded the declarations separately in the submission system (<https://journals.plos.org/plosone/s/submission-guidelines#loc-additional-information-requested-at-submission>). However, we have included the key declarations here for the reviewer:  ***Ethics approval and consent to participate***  *Ethics approval was obtained from the Ethics Committee of the University of Heidelberg (registration number S-854/2020). All methods were carried out in accordance with relevant guidelines and regulations. Informed consent was waived by the Ethics Committee of the University of Heidelberg due to retrospective nature of the study.*  ***Competing interests***  *The authors declare that they have no competing interests.*  ***Funding***  *No funding was received to conduct this study* |
| The terms “prevalence” and “proportion” are used interchangeably in the document. Please choose one and use it consistently. | We agree with the reviewer that we have used these terms inconsistently. We have revised the manuscript carefully and replaced the term “prevalence” with the term “proportion” where appropriate. |
